# Co-occurrence of Anomalous Right Coronary Artery Origin and Subaortic Membrane in an Adult Male

**DOI:** 10.7759/cureus.27110

**Published:** 2022-07-21

**Authors:** Olushola O Ogunleye, Oluwafemi Ajibola, Mehmood Cheema, Busala Oke, Jason Sperling

**Affiliations:** 1 Department of Internal Medicine, Vassar Brothers Medical Center, Poughkeepsie, USA; 2 Division of Cardiovascular Surgery, Vassar Brothers Medical Center, Poughkeepsie, USA

**Keywords:** cardiac surgery, congenital heart anomalies, subaortic membrane, right coronary artery, anomalous origin

## Abstract

Anomalous origin of the right coronary artery (ARCA) represents <3% of congenital coronary anomalies, while the subaortic membrane represents 6.5% of congenital heart anomalies. Symptomatic co-occurrence of ARCA and subaortic membrane in an adult is rare. A 68-year-old man developed a non-ST-elevation myocardial infarction necessitating percutaneous coronary intervention (PCI) four years prior to presentation at our hospital. In the years after his PCI, he developed progressive exertional breathlessness. Following a positive treadmill EKG, he underwent coronary CT angiography that indicated RCA dominance with ARCA arising from the left coronary sinus and coursing between the ascending aorta and pulmonary artery, causing 50-60% intraluminal narrowing at rest without atherosclerotic plaque. Echo showed normal left ventricular ejection fraction (LVEF) and a surprise finding of the subaortic membrane, with a modest gradient. He underwent successful resection of the subaortic membrane and unroofing of the anomalous RCA tunnel with tract marsupialization. The post-operative period was complicated by arrhythmias necessitating electrical cardioversion. At discharge, he was sent home on apixaban, bisoprolol, aspirin, atorvastatin, and an amiodarone taper. The subaortic membrane would not have required intervention independently because it was not associated with a severe gradient. However, surgery is recommended for symptomatic ARCA or subaortic membrane; hence, our patient underwent surgical management. Atrial fibrillation and flutter are the most common arrhythmias following cardiac surgery. Due to the patient’s increased risk of complications, cardioversion and anticoagulation were pursued. Although ARCA is congenital, our patient had been asymptomatic for most of his life, suggesting that the development of the subaortic membrane might have triggered symptom onset, combining a modest subaortic gradient with previously asymptomatic exercise-induced right coronary ischemia. Clinicians should consider evaluating for secondary structural heart conditions in newly symptomatic adult patients with ARCA due to the risk of sudden cardiac death, to provide the most complete treatment.

## Introduction

Several forms of anomalous coronary origin have been reported in the literature [1,2]. Anomalous origin of the right coronary artery (ARCA) from the left sinus of Valsalva and running between the aorta and pulmonary artery represents less than 3% of congenital coronary anomalies and 0.025% to 0.25% of all congenital abnormalities [1]. Most patients with ARCA are asymptomatic [1,3]. 

A subaortic membrane is a crescent-shaped fibrous or muscular membrane that forms just below the aortic valve, obstructing the left ventricular outflow tract (LVOT) [4,5]. The subaortic membrane is the most common type of sub-valvular aortic stenosis (75-85% of cases) [4,5]. It is the second most common cause of aortic stenosis, represents 6.5% of all congenital heart abnormalities, and can occur alone or in combination with other congenital cardiac abnormalities [4]. Although it was previously considered to be exclusively a congenital childhood disease, the occurrence of an acquired subaortic membrane has been reported in adults [6-9].

During our literature review, we did not find any prior case reports of ARCA occurring with the subaortic membrane in an adult. Hence, we present the case of a middle-aged man presenting with symptoms attributed to the co-occurrence of ARCA and subaortic membrane.

## Case presentation

**Figure 1:**
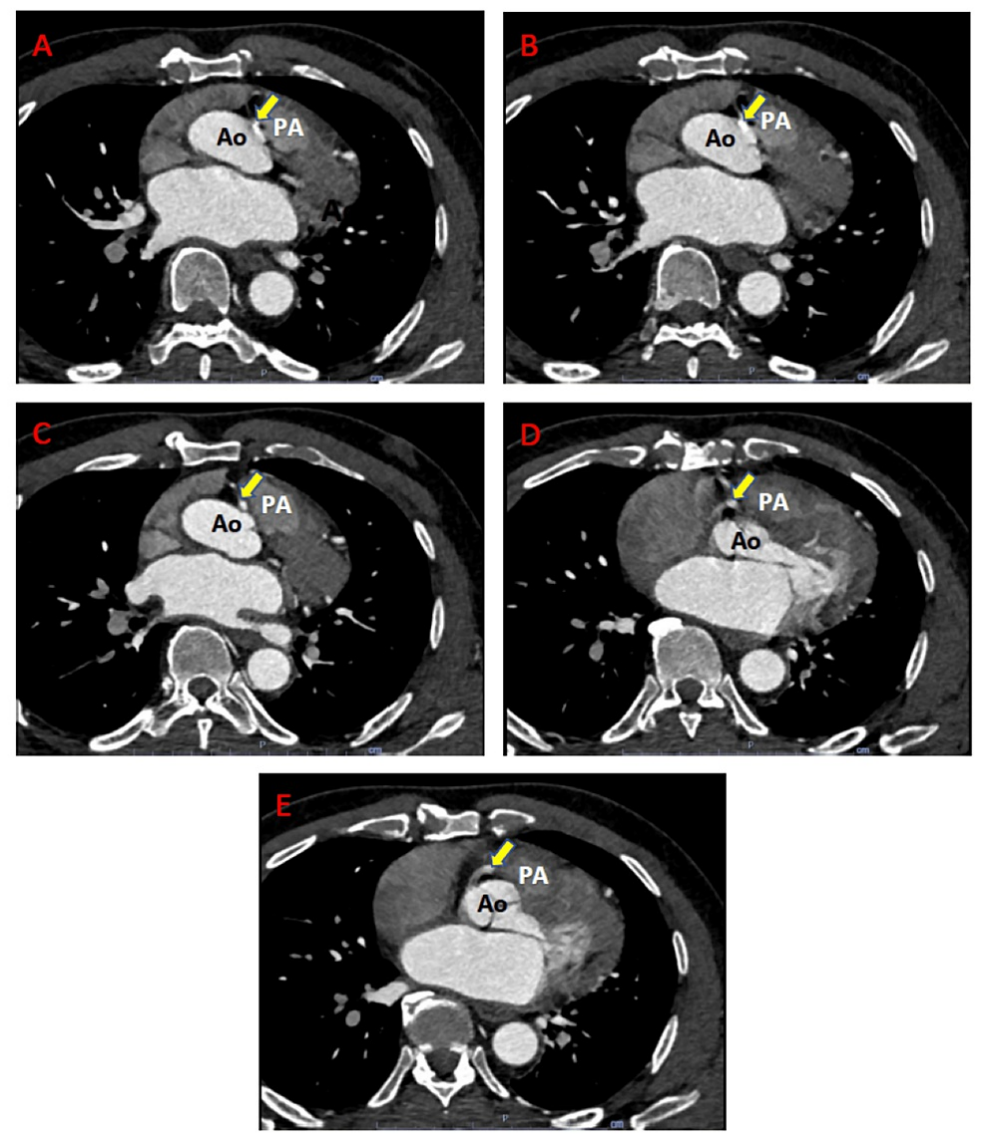
Coronary CT angiogram. (A)-(E) Transverse slices of a coronary CT angiogram (in craniocaudal direction) showing the origin of the RCA (yellow arrow) from the left sinus of Valsalva and its course between the ascending aorta (Ao) and main pulmonary artery (PA).

A 68-year-old male never-smoker with a history of hypertension, hyperlipidemia, sinus bradycardia, and coronary artery disease presented to our hospital for elective cardiothoracic surgery following a diagnosis of ARCA in the outpatient setting. The patient had been active as a recreational soccer player for most of his adult life without any episodes of exertional chest pain. However, four years prior to this presentation, he developed sudden-onset cardiac chest discomfort and was diagnosed with non-ST-elevation myocardial infarction. At the time, he underwent left heart catheterization with drug-eluting stent placement for a 90% stenotic lesion in the proximal left anterior descending artery (LAD). During the procedure, an anomalous RCA takeoff was suspected because there was some difficulty selectively engaging his RCA, but this was not investigated further since there was no evidence of myocardial ischemia within the RCA territory at the time. The patient subsequently had mild improvement in exercise tolerance, but progressively became more breathless with activity. An echocardiogram done six months prior to this presentation indicated that he had normal left ventricular function with an ejection fraction of >55% and no significant LVOT gradient (peak gradient of 10.9 mmHg), but there was an unexpected finding of a subaortic membrane. Two months prior to this presentation, he underwent a treadmill stress EKG test that was limited by fatigue, shortness of breath, and dysrhythmias without chest pain. Consequently, he had coronary CT angiography (Figure 1A-1E), which indicated RCA dominance and confirmed the anomalous RCA origin from the left coronary sinus with an inter-arterial course between the ascending aorta and pulmonary artery, causing 50-60% intraluminal narrowing without atherosclerotic plaque. He underwent pre-surgery workup, including cardiac catheterization, that showed patency of his prior LAD stent without any other angiographically significant coronary artery disease.

**Figure 2:**
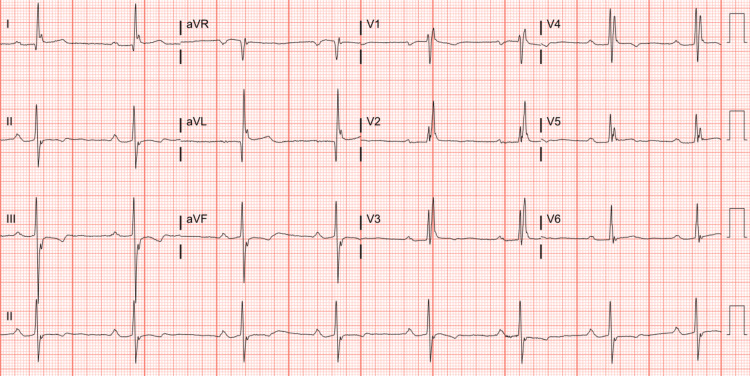
Pre-operative ECG. Pre-operative ECG showing sinus bradycardia with first-degree AV block.

At the presentation for elective surgery, resting EKG showed sinus bradycardia with first-degree AV block and no significant ST-segment abnormalities (Figure 2). He underwent successful resection of the subaortic membrane and unroofing of the anomalous RCA tunnel with tract marsupialization. Intraoperative findings included an intramural (aortic) tunnel of the RCA from the left sinus, arising distinctly just above the left main ostium, then tracking behind and above the top of the right/left aortic valve commissure. To prevent collapse and obstruction, the tract edges were marsupialized to the aorta internally, and the commissure was re-suspended after marsupialization.

**Figure 3:**
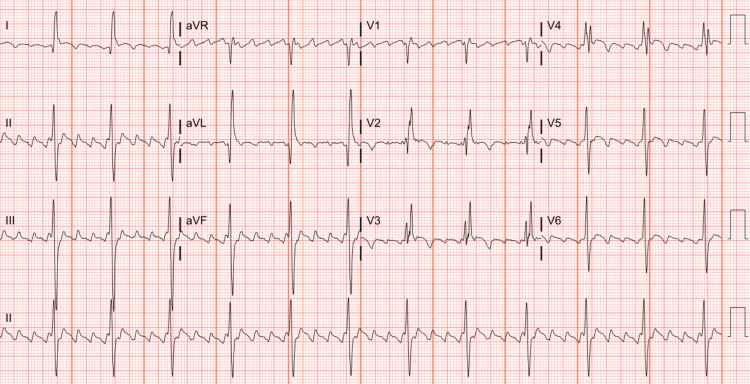
ECG on post-operative day four. ECG showing atrial flutter with 4:1 conduction on post-operative day four.

**Figure 4:**
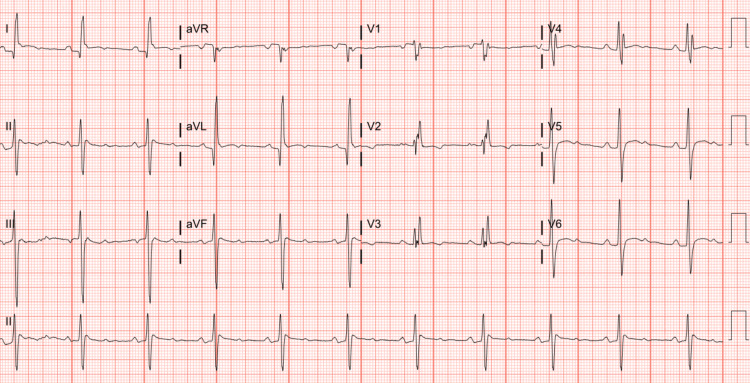
ECG after cardioversion on post-operative day six. ECG showing restoration of normal sinus rhythm after successful direct current cardioversion.

The patient’s post-operative course was complicated by transient complete heart block a few hours after surgery. He developed atrial fibrillation on post-operative day three, then 4:1 atrial flutter on post-operative day four (Figure 3), necessitating commencement on amiodarone and heparin drips, then transitioned to oral amiodarone 400 mg twice daily. After failing pharmacologic cardioversion, he underwent successful direct current cardioversion on post-operative day six. He subsequently remained hemodynamically stable and in sinus rhythm (Figure 4). On post-operative day seven, he was discharged home on apixaban, bisoprolol, aspirin, atorvastatin, and an amiodarone taper. He was scheduled for outpatient follow-up with cardiothoracic surgery within one to two weeks after discharge. He was also asked to follow up within two to four weeks of discharge with the non-invasive cardiologist, the electrophysiologist, and his primary care physician.

## Discussion

Coronary artery anomalies often remain undiagnosed unless they are symptomatic. In a study in which 18,950 autopsies were performed, coronary artery anomalies were reported in only 0.3% of cases [10]; whereas in another study involving 1,686 patients that underwent coronary arteriography for symptoms suggestive of cardiac ischemia, coronary artery anomalies were reported in 1.3% of cases [11]. Sites of anomalous origin of coronary arteries include the contralateral coronary artery, contralateral coronary sinus, left ventricle, pulmonary arteries, bronchial arteries, subclavian arteries, carotid arteries, internal mammary arteries, and innominate artery [3]. Congenital heart conditions commonly associated with subaortic membrane include ventricular septal defect, coarctation of the aorta, atrial septal defect, patent ductus arteriosus, bicuspid aortic valve, and double-outlet right ventricle [6]. Although approximately 23% of patients with coronary artery anomalies also have other congenital cardiovascular anomalies [10], subaortic membranes are rarely associated with coronary artery anomalies.

In the current literature, there are no known reports of concurrent ARCA and subaortic membrane in an adult. Most patients with ARCA and most patients with subaortic membrane are asymptomatic; hence, many cases remain undiagnosed. In patients with ARCA, the severity of restricted blood flow is related to symptomatology. When symptomatic, ARCA can present with dyspnea, angina, arrhythmia, or sudden cardiac death (SCD) [1,12]. The subaortic membrane usually remains asymptomatic until when it progresses to LVOT obstruction or becomes complicated by aortic insufficiency [5,7,9,13,14]. The symptomatic subaortic membrane can be present with dyspnea, palpitations, chest pain, syncope, new diastolic murmurs, or signs of left ventricular hypertrophy [14,15]. As seen in our patient, the co-occurrence of these two conditions increases the risk of dyspnea, activity limitations, arrhythmias, myocardial ischemia, or sudden death.

Although ARCA is congenital, our patient had been asymptomatic for most of his life. Unlike most other congenital heart diseases, the subaortic membrane does not appear during the embryonic period and is very rarely found in neonates or infants; rather, most cases of subaortic membrane manifest during the first decade of life [4,6]. An explanation for the acquired subaortic membrane is increased turbulence at the LVOT from underlying cardiac disease that increases septal shear stress, producing adaptive changes in cell proliferation and leading to the formation of the membrane [14]. In our patient, the turbulent flow created by the abnormal path of the ARCA might have predisposed to the development of the subaortic membrane. Along with advancing age, the modest subaortic membrane might have contributed to an increase in pulmonary artery pressures over time and triggered symptom onset, resulting in exercise-induced right coronary ischemia.

Factors that contribute to coronary blood flow restriction in patients with ARCA include the acute angle at which the ARCA arises, the narrow (slit-like) orifice at its origin, compression of the intramural segment of the ARCA by the aortic valve commissure, and compression of the ARCA as it courses between the aorta and the pulmonary artery [12]. Some autopsy-based studies report that the slit-like orifice and the acute takeoff angle occur commonly in patients who had SCD [12].

ARCA is often diagnosed with multidetector coronary CT angiography or conventional chest CT angiography in patients undergoing evaluation for coronary artery disease, but may occasionally be diagnosed via echocardiography in symptomatic patients [2,3]. Coronary CT angiography is more sensitive and more favored than echocardiography for the diagnosis of ARCA [2], but echocardiography has good sensitivity for the diagnosis of a subaortic membrane [5]. The cornerstone of treatment for symptomatic patients with ARCA and subaortic membrane remains surgery, while conservative management is preferred for asymptomatic patients [1]. Hence, our patient underwent resection of the subaortic membrane and unroofing of the ARCA tunnel with tract marsupialization.

Arrhythmias occur frequently after cardiac surgery, the most common of which are atrial fibrillation and atrial flutter [16,17], as seen in our patient. Post-operative atrial fibrillation (POAF) occurs in about 25-40% of patients following coronary artery bypass graft (CABG) surgery, while at least half (50-60%) of patients who get valvular surgery develop POAF [16,17]. The exact mechanism of arrhythmias following cardiac surgery is not known, but it is probably multifactorial [16]. Postulated mechanisms include ectopic beats that originate from the thoracic veins or the pulmonary veins, as with atrial fibrillation that occurs in non-surgical patients [16]. However, most episodes of POAF are suspected to be triggered by premature atrial contractions arising from a conductive atrial site [16]. The intensified post-surgical sympathetic response following cardiac surgery might contribute to the increased risk of arrhythmias overall. As seen in our patient, up to 70% of initial episodes of POAF tend to occur within the first four days after cardiac surgery, while up to 60% of recurrences occur within two days of the initial episode [16]. Due to the increased risk of further complications, such as congestive heart failure, ischemic stroke, prolonged hospital stay, and rehospitalization, it was necessary to cardiovert the patient and start him on anticoagulation prior to discharge [16,17].

## Conclusions

There are no known prior case reports of co-occurring ARCA and subaortic membrane in adults. This report highlights the co-occurrence of these two conditions in a middle-aged man who had previously been tolerating moderate-to-high-intensity physical activity. The subaortic membrane was probably acquired in the setting of the patient’s underlying cardiac disease and increased turbulence at the LVOT. Although ARCA is a congenital anomaly, the patient had been asymptomatic for most of his life. This suggests that the association of ARCA with the subaortic membrane might have served as the catalyst to cause symptom onset. To provide the most complete treatment, clinicians should consider evaluating for secondary structural heart conditions in newly symptomatic adult patients with ARCA due to the risk of sudden cardiac death.
